# Shadows of anyons and the entanglement structure of topological phases

**DOI:** 10.1038/ncomms9284

**Published:** 2015-10-06

**Authors:** J. Haegeman, V. Zauner, N. Schuch, F. Verstraete

**Affiliations:** 1 Department of Physics and Astronomy, University of Ghent, Krijgslaan 281 S9, B-9000 Ghent, Belgium; 2 Vienna Center for Quantum Technology, University of Vienna, Boltzmanngasse 5, 1090 Wien, Austria; 3 JARA Institute for Quantum Information, RWTH Aachen University, D-52056 Aachen, Germany

## Abstract

The low-temperature dynamics of quantum systems are dominated by the low-energy eigenstates. For two-dimensional systems in particular, exotic phenomena such as topological order and anyon excitations can emerge. While a complete low-energy description of strongly correlated systems is hard to obtain, essential information about the elementary excitations is encoded in the eigenvalue structure of the quantum transfer matrix. Here we study the transfer matrix of topological quantum systems using the tensor network formalism and demonstrate that topological quantum order requires a particular type of ‘symmetry breaking' for the fixed point subspace. We also relate physical anyon excitations to domain-wall excitations at the level of the transfer matrix. This formalism enables us to determine the structure of the topological sectors in two-dimensional gapped phases very efficiently, therefore opening novel avenues for studying fundamental questions related to anyon condensation and confinement.

## Abstract (web summary)

Tensor network states efficiently parametrize many-body quantum ground states and entanglement properties of strongly correlated systems. Here, the authors show how the presence of anyons and topological order can be related to symmetry breaking in the virtual boundary theory of the network.

## Graphical abstract

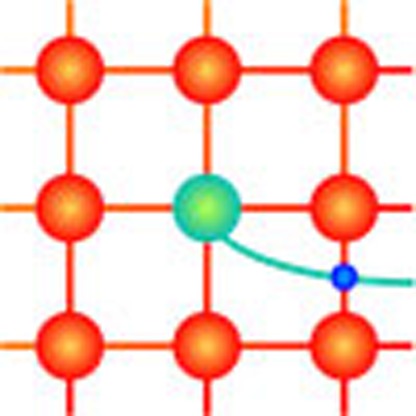

One of the major recent advances in the understanding of strongly correlated quantum many body systems has been the investigation of quantum entanglement in terms of area laws^1^,^2^, the entanglement spectrum^3^ and the associated entanglement Hamiltonian^4^,^5^. The structure of entanglement in gapped quantum systems has resulted in the powerful parameterization of quantum ground states in terms of so-called tensor network states, such as matrix product states (MPS)^6^,^7^ or their higher dimensional analogues, projected entangled pair states (PEPS)^8^,^9^. In a translation invariant tensor network state, the entanglement features can be extracted from the leading eigenvector of the so-called (quantum) transfer matrix, which naturally appears whenever the quantum state is mapped to a classical partition function (using for example, a Trotter decomposition) and then sliced along the virtual direction, that is, the Trotter direction or direction of imaginary time^10^. In a very recent publication^11^, we have observed that the other eigenvalues of the transfer matrix also contain useful information, thst is, they contain crucial information about the elementary excitations and the corresponding dispersion relations of the system. This is, in many ways, surprising as this information is completely encoded in the ground-state description of the system, *a priori* without any information about the original Hamiltonian for which it was the exact or approximate ground state.

This relation is extremely useful for the case of two-dimensional systems, where systematic methods to extract the dispersion relation of elementary excitations are virtually non-existent. This was illustrated in ref. 11 by studying the Affleck–Kennedy–Lieb–Tasaki model^12^,^13^. However, for the case of two-dimensional quantum spin systems, exhibiting topological quantum order, the excitation spectrum can be much richer. In contrast to trivial phases, where the elementary excitations can provably be created as Bloch waves of localized perturbations^14^, elementary excitations in topological phases are typically anyons that come with ‘strings attached'. Note that topologically non-trivial excitations also appear in one-dimensional systems, such as in the Lieb–Liniger model^15^ or as a domain-wall excitations in systems with discrete symmetry breaking. In terms of MPSs, those excitations can be represented with a Bloch like ansatz where an extra half-infinite string operator is attached to the local perturbation^16^,^17^,^18^,^19^.

The PEPS representation of topologically ordered ground states, either as variational approximation^20^,^21^ or as exact description of the Levin–Wen model wave functions^22^,^23^,^24^, has been well established. Here we study the full spectrum of the transfer matrix of topologically ordered PEPS^5^ on the infinite plane or cylinder. Much like the prisoners in Plato's cave, we observe one-dimensional domain walls in the spectrum of the transfer matrix as shadows of the true anyons in the two-dimensional world. By clarifying how the different anyon sectors are manifested at the virtual level, we can probe the dispersion relation of single anyon states. We discuss how the presence of anyons, and thus of topological order, requires a particular type of symmetry breaking in the fixed point subspace of the transfer matrix and how anyon condensation or confinement^25^,^26^ is reflected in these virtual description. We illustrate our results by studying the PEPS description of the toric code model with string tension and the resonating valence bond state. Our results also confirm that we can construct approximate eigenvectors of PEPS transfer matrices using the matrix product ansatz developed for one-dimensional quantum Hamiltonians in refs 17, 27.

## Results

### Topological order in PEPS

**Figure 1:**
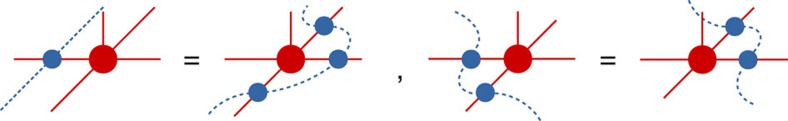
Topological order in PEPS. When representing the state of a quantum lattice system as a PEPS, a local tensor (red) with four virtual indices (which are contracted in the network) and one physical index (pointing upwards) is associated to every site. The PEPS description of topologically ordered states are characterized by the existence of string operators living at the virtual level of the network (acting only on the contracted indices) and that can be pulled through the lattice, such that they are locally invisible. When the string operators are expressed as MPOs, the pulling through yields a local symmetry condition between the PEPS tensor (red) and the MPO tensors (blue), which characterizes the global topological order in the full-quantum state.

The convience of the tensor network description of quantum states is that the global, topological properties of the state are reflected in the symmetries of the local tensors. Since topological phases are not characterized by local order parameters, these symmetries act purely on the virtual levels of the tensors. In particular, it was recently established that topological order in PEPS can be characterized by the existence of matrix product operators (MPO), which can be pulled through the lattice at the virtual level (see Fig. 1)^28^,^29^,^30^. Closed MPO loops around a topologically trivial region define the invariant subspace on which the PEPS tensors are supported and in this way characterizes the topological properties of the state, such as the topological corrections to the entanglement entropy. They act as virtual operators *O*_*i*_ and satisfy a fusion algebra 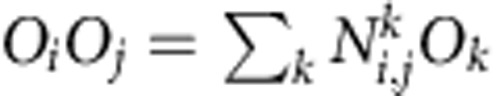. Indeed, as shown in ref. 30, for the case of the Levin–Wen string net models, the different MPOs *O*_*i*_ can be associated to and labelled by the different string types *i*=1,…,*N* of the input category that defines the string net model. A PEPS in a trivial phase is characterized by a single MPO *O*_1_ that acts as the identity in the relevant subspace. Another interesting case is that of the quantum double models, which can be described using G-injective PEPS^28^. This is a special case of the formalism of ref. 30 where the MPOs are labelled by the group elements *g*∈*G* and correspond to representations *O*_*g*_=*U*(*g*) of the group action at the virtual level. The pulling-through condition is satisfied since the tensors are only supported on the invariant subspace defined by the projector *P*=∑_*g*_*O*_*g*_.

While the pulling-through conditions ensure that the presence of an MPO string cannot be detected locally, noncontractible MPO loops can have global effects, such as adding a non-trivial flux in the system, and can, therefore, be used to map one ground state to another one. The relevance of these virtual MPOs is that also away from the the renormalization group fixed point—where the physical string operators are spread out and not exactly known^31^—the MPOs at the virtual level remain strictly local and the ‘pulling-through' symmetry of the PEPS tensor is exactly preserved.

### Anyon excitations in the PEPS picture

Having a translation invariant PEPS description of the ground state of a topological phase, one can easily argue that a suitable ansatz to model single anyon excitations is obtained by modifying the ground-state tensors in a local region (for example, a single site) and attaching a half-infinite string to it, which is exactly given by this MPO at the virtual level. The MPO will give rise to the non-trivial braiding statistics of these excitations, while the ‘pulling-through' assures that the bulk of the string is locally unobservable, so that the energy density is left at its ground-state value sufficiently far away from the end point. Away from the renormalization group fixed point, these excitations will disperse and a proper eigenstate can be obtained by building a momentum superposition with the momentum *k*_*x*_ and *k*_*y*_ in the *x* and *y* direction.

**Figure 2:**
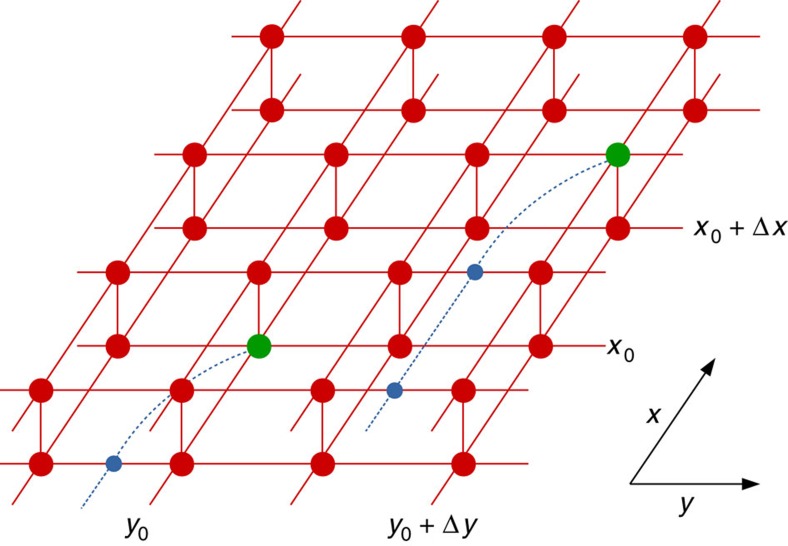
Overlap of topologically non-trivial PEPS excitation. A PEPS ansatz for a topologically non-trivial excitation is obtained by replacing a single tensor of the PEPS ground state (red) at position (*x*_0_,*y*_0_) with a perturbed tensor (green), which has a virtual MPO string (blue) attached. Away from a renormalization group fixed point, excitations will disperse but translation invariance can be exploited to build a reliable excitation ansatz from a momentum superposition of the local perturbation. As argued in ref. 11, the variational excitation energy will depend on the overlap of these states, which consist of terms where the string end points in ket and bra are separated by any difference (Δ*x*,Δ*y*) and which carry a momentum factor *exp*(i*k*_*x*_Δ*x*+i*k*_*y*_Δ*y*) accordingly. For momenta where these terms interfere coherently, a minimum in the corresponding variational dispersion relations is observed.

Note that the topological quantum numbers of the anyon excitations are not completely specified by the string type *i*, but are determined by the structure of the excitation tensor in the ansatz of Fig. 2. For the case of the quantum doubles, it has been shown that the string type corresponds to the magnetic flux, whereas the charge quantum number is determined by the representation space on which the local tensor is supported^28^. A complete characterization of the different anyon sectors in the PEPS formalism would take us to far and is presented elsewhere (N. Bultinck, M. Mariën, D. Williamson, J. Haegeman, F. Verstraete, manuscript in preparation).

### Transfer matrix: symmetries and domain walls

**Figure 3:**
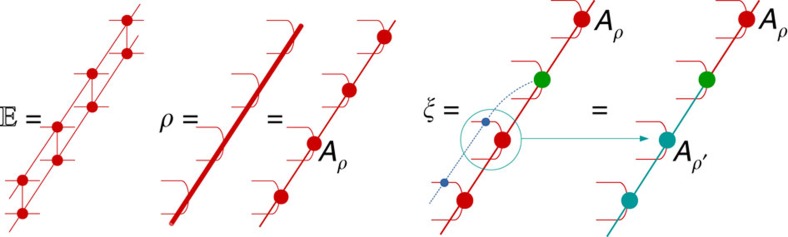
Transfer matrix and boundary states. For evaluating the overlap of Fig. 2, we first define the (normal) transfer matrix 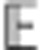. The contribution of the infinite number of transfer matrices to the right of *y*_0_+Δ*y* is captured in a boundary state *ρ*, which corresponds to one of the fixed points of 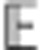 and is represented as a MPS with tensors *A*_*ρ*_ (big red circle). When contracting the additional column of Fig. 2 at position *y*+Δ*y* containing the excitation, the boundary state is transformed into a topologically non-trivial state *ξ*, which differs from the fixed point *ρ* by a local perturbation (green) and a half-infinite string (blue). The effect of this string is to transform the MPS tensors *A*_*ρ*_ into the MPS tensors *A*_*ρ*′_ (cyan) of a different fixed point *ρ*′ of the transfer matrix 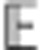. The boundary state can thus be reinterpreted as a domain-wall excitation of the transfer matrix, which interpolates between two different fixed points. We can expand it into a full basis of topologically non-trivial eigenvectors of 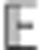 with specified momentum *k*_*x*_. Coherent interference of the different terms in Fig. 2 is obtained when the momentum *k*_*y*_ aligns with (minus) the phase of one of the eigenvalues of the transfer matrix with large magnitude.

As in ref. 11, one can now argue that the dominant contribution to the variational dispersion relation is coming from the normalization of these states, which is given by the sum of overlaps of ket and bra with string end points at different positions (*x*_0_, *y*_0_) and (*x*_0_+Δ*x*, *y*_0_+Δ*y*), as illustrated in Fig. 2. If we orient the strings along the *x* direction and first contract the tensor network along the *y* direction, the central object will be the transfer matrix 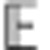 in the *x* direction, as defined in Fig. 3. The pulling-through condition of Fig. 1 ensures that 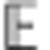 commutes with infinite MPO strings along the *x* axis in the ket and bra level separately. We thus obtain 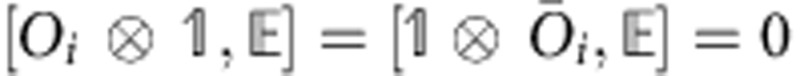, ∀*i*=1,…, *N* where *O*_*i*_ now denotes an infinite MPO of type *i* along the *x* axis. Normalization of the PEPS ground state requires that the largest eigenvalue of 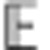 is 1, and the infinite power of the transfer matrix 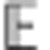 in the overlap of Fig. 2 in the regions *y*<*y*_0_ and *y*>*y*_0_+Δ*y* can be replaced by its left and right fixed point *σ* and *ρ*, which we represent as an infinite MPS with matrices *A*_*σ*_ and *A*_*ρ*_ (Fig. 3).

The transfer matrix 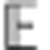 can have a degenerate fixed point structure, since for a given right fixed point *ρ*, one can build other fixed points 
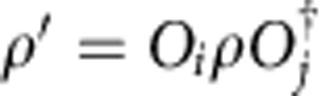
 for all *i*, *j*=1,…, *N*. One could expect that this generally gives rise to an *N*^2^-dimensional fixed point subspace. However, at the renormalization group fixed point of the topological phase, we can easily check that the fixed point subspace of 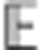 is exactly spanned by *ρ*_*k*_=*O*_*k*_ for *k*=0,…, *N*, and is thus only *N*-dimensional. The degeneracy and labelling of the fixed point subspace remains intact throughout the topological phase, even though *ρ*_*k*_ will no longer exactly equal *O*_*k*_. This implies, in particular, that 
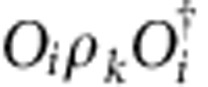
 can be expanded into a linear combination of ∑_*l*_*c*_*kl*_*ρ*_*l*_ with *c*_*k*,*k*_=1, compatible with the fact that fusing 
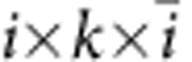
 will have a fusion channel *k*. We now argue why this property is required to support anyonic excitations with a half-infinite virtual string of type *i*.

Contracting the tensor network in Fig. 2 from right to left up to position *y*_0_+Δ*y* gives rise to some boundary state *ρ* in the fixed-point subspace, whose precise choice is set by the boundary conditions at *y*=+∞. The topological invariance ensures that this choice has no effect on local expectation values. As we now further contract from right to left, we pass the position *y*_0_+Δ*y* containing the excitation in the bra level. Here the boundary state is perturbed locally at *x*=*x*_0_+Δ*x*. In addition, it will be acted on by a half-infinite MPO string of type *i*, which has the effect of changing the MPS tensors from *A*_*ρ*_ to *A*_*ρ*′_ with 
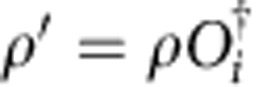
 *x*<*x*_0_+Δ*x*. At the level of the transfer matrix, the boundary state now takes the form of a domain wall interpolating between the two different fixed points. Because of translation invariance, all overlaps corresponding to the momentum superposition in the *x* direction can be summed and the resulting state takes the form of a topologically non-trivial state with momentum *k*_*x*_, similar to the domain-wall excitation ansatz used for one-dimensional Hamiltonians in ref. 17. We thus have to consider the spectrum of topologically non-trivial eigenstates *ξ*_*j*_ (Fig. 3) of 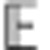 with momentum *k*_*x*_. Indeed, further contracting up to *y*_0_ yields the *k*_*x*_-dependent eigenvalues *λ*_*j*_(*k*_*x*_) of *ξ*_*j*_ to some power Δ*y*, which dictates the *k*_*y*_ dependence of the dispersion relation of the corresponding physical excitation.

At point *y*_0_, the boundary state is acted on with a second half-infinite string (also with momentum *k*_*x*_ in the *x* direction), now in the ket level. After that, the state is collapsed onto the topologically trivial left fixed point *σ*. To have a non-vanishing overlap, the corresponding boundary state—with half-infinite strings in both the ket and bra level—should have a contribution in the trivial sector. This will be true if the property stated above is satisfied, that is, 
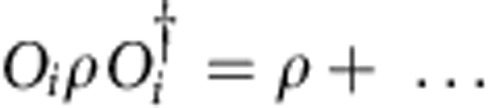
 for all *ρ*.

As we perturb the state out of the topological phase, there are two ways in which the existence of topologically non-trivial excitations can break down. If the transfer matrix 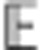 has a unique (maximally ‘symmetric') fixed point, which is invariant under the action of any string *O*_*i*_, then the states *ξ* interpolate between the same fixed point left and right and are thus indistinguishable from topologically trivial local perturbations (without string). This scenario is realized when the corresponding anyon has condensed into the ground state^25^,^26^. A second possibility is that the fixed-point structure of 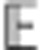 has an even larger ‘symmetry breaking', in such a way that the state with half-infinite strings in ket and bra level is still completely topologically non-trivial and has zero overlap with the left fixed point *σ*. More specifically, the fidelity per site, defined as the largest eigenvalue *λ* of 
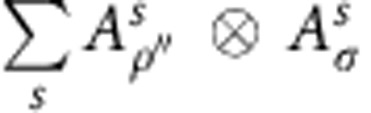
 with 
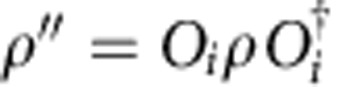
, satisfies |*λ*|=e^−*t*^<1 and the normalization of the state goes down as e^−*tL*^ with *L* the length of the strings. Hence, the non-zero value of *t* acts as a string tension and only bound states of two excitations connected by a finite string can exist, corresponding to the mechanism of confinement.

In the generic case, there can of course be several transitions corresponding to, for example, the condensation of only some anyonic sectors and the induced confinement of other anyon sectors. Note that, since topological phase transitions correspond to symmetry breaking phase transitions at the virtual level of the PEPS description, we can also find virtual order parameters, as illustrated in the toric code example. A more in-depth study of these aspects of anyon condensation within the framework of PEPS will be provided elsewhere (J. Haegeman, N. Schuch, F. Verstraete, manuscript in preparation).

### Mixed transfer matrix and momentum fractionalization

**Figure 4:**
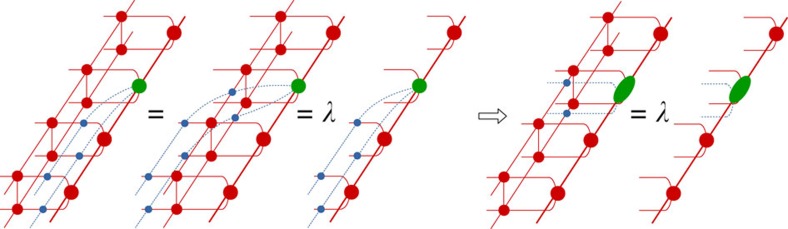
Mixed transfer matrix. Using the pulling-through property of the virtual string, we can transfer the eigenvalue equation for topologically non-trivial excitations of the transfer matrix into a normal eigenvalue problem for a so-called ‘mixed' transfer matrix. The mixed transfer matrix differs from the normal transfer matrix as it is threaded by an MPO string in ket and bra (which can be the identity) and, therefore, acts on a larger space corresponding to the two additional MPO indices. This identification is useful when putting the system on a long cylinder instead of on the infinite plane, as it allows to use periodic boundary conditions in the *x* direction.

In the above discussion, we have explained how information about anyon excitations in topological phases can be obtained from the topologically non-trivial excitations of the translation invariant transfer matrix, which has a degenerate fixed point subspace in the case of topological order. Fig. 4 motivates an alternative approach. By using the pulling-through property of the MPO, we can rewrite the eigenvalue equation for a topologically non-trivial excitation of 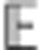 as a normal (topologically trivial) eigenvalue problem for a so-called mixed transfer matrix. The latter is threaded by an MPO string and is thus defined on a larger vector space corresponding to the presence of additional MPO indices. Physically, we are effectively rotating the MPO strings attached to the anyon excitation to lie along the *y* direction.

**Figure 5:**
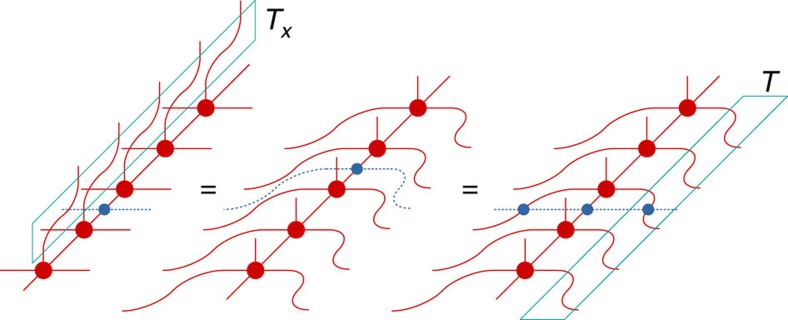
Virtual representation of the translation operator. When the system is studied on a long cylinder, translations around the cylinder will also affect the string of the topological excitations, which thus leads to a non-trivial representation *τ* of the translation operator on the virtual level. Consequently, the mixed transfer matrix defined in Fig. 4 will be invariant under the action of 
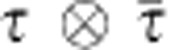
 and its spectrum can be labelled with the corresponding momentum quantum numbers. Due to the non-trivial interaction with the string, this can give rise to momentum fractionalization.

By doing so, we can make the *x* direction finite and periodic, which allows to work on a cylinder with finite circumference. The fixed points of these mixed transfer matrices were first studied in ref. 5. In this case, physical translations in the *x* direction have a representation as modified translation operators at the virtual level of the transfer matrix with a non-trivial action on the extra MPO indices (see Fig. 5). This results in a momentum label that can have a fractional discretization in the circumference of the cylinder, similar to what is observed in the case of momentum polarization^32^. We elaborate on this aspect in Supplementary Note 1 and Supplementary Figs 1 and 2.

### Toric code model with string tension

Let us now illustrate this approach using the toric code ground state, to which we apply a local filtering


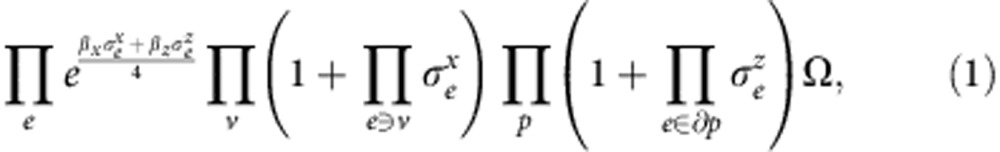


with |Ω〉 the fully polarized spin state 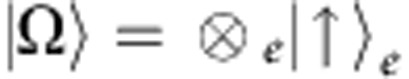. The filtering induces dynamics to the elementary excitations of the toric code ground state and can drive the system into a trivial phase. Along the coordination axes *β*_*x*_=0 or *β*_*z*_=0, it can be interpreted as string tension in either the group or representation basis and can be solved exactly^33^,^34^. The full two-dimensional phase diagram as a function of *β*_*x*_ and *β*_*z*_ was studied in ref. 35 using a fidelity approach^36^,^37^,^38^.

The PEPS representation of the toric code ground state was first constructed in ref. 39 and its properties were discussed at length in ref. 28. The PEPS tensors satisfy the property of G-injectivity, where in this case 
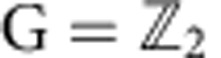
. For this particular case, it means that the MPO projector *P* is given as *O*_0_+*O*_1_=^⊗*L*^+*Z*^⊗*L*^, with *L* the length of the MPO string and *Z* a representation of the non-trivial element of 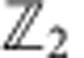. Correspondingly, the transfer matrix 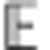 along the *x* direction has the global symmetry 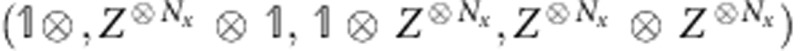 with *N*_*x*_ the number of sites in the *x* direction. The filtering operation is applied at the physical level of the PEPS and has no effect on any of these properties. It does, however, influence the manifestation of the symmetry in the fixed point subspace of the transfer matrix.

**Figure 6:**
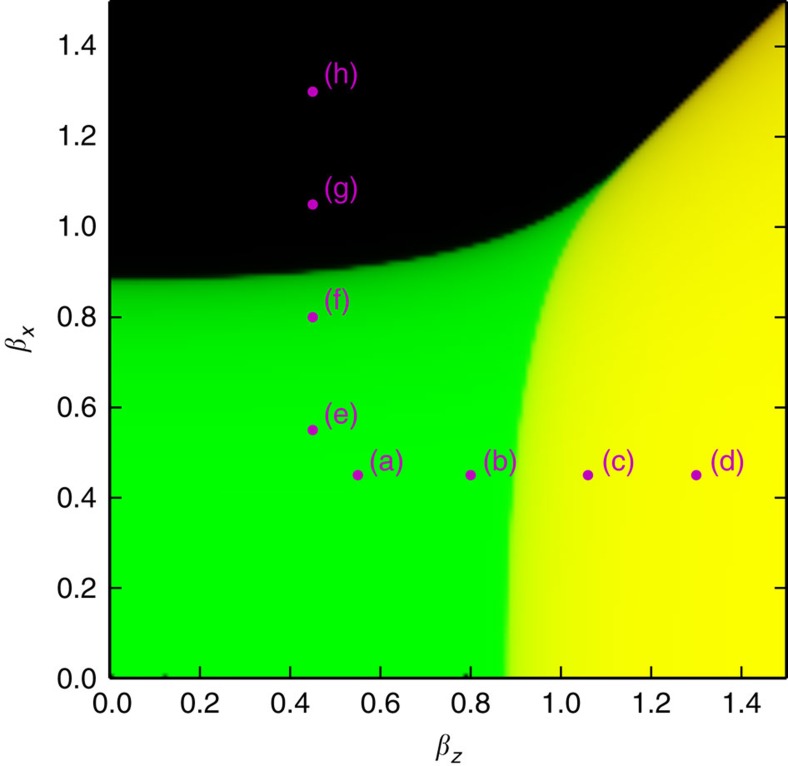
Phase diagram of the filtered toric code. The phase diagram of the filtered toric code in Equation (1) can be obtained by measuring the expectation value of the virtual order parameters. The expectation value of *X*⊗*X* is used as green intensity and of *X*⊗ as red intensity. If both are zero (black region) the transfer matrix has a unique symmetric fixed point corresponding to a trivial phase where the string-like excitations (flux) are condensed and charges are confined. If *X*⊗*X* is non-zero but *X*⊗ is zero (green region), the transfer matrix fixed-point subspace is two-dimensional and breaks the 
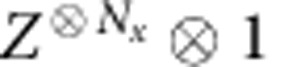
 symmetry but is symmetric under the action of 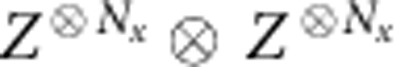 corresponding to the topological phase with anyon excitations. If both expectation values are non-zero (note that green and red combine to the yellow region), the transfer matrix fixed point subspace is four-dimensional and breaks the complete symmetry group. This corresponds to a trivial phase in which the string-like excitations (flux) are confined and charges are condensed. Points (a) to (h) indicate the parameter combinations for the different spectra presented in Fig. 7.

At the virtual level of the PEPS, we can use local order parameters *X*⊗ and *X*⊗*X* to detect the symmetry breaking of *Z*⊗*Z* and *Z*⊗, respectively, in the fixed-point subspace, where *X* is an operator such that *XZ*=−*ZX*. Fig. 6 shows the structure of the fixed point subspace. The topological ‘toric code' phase is characterized by a doubly degenerate fixed point, which is invariant under the subgroup 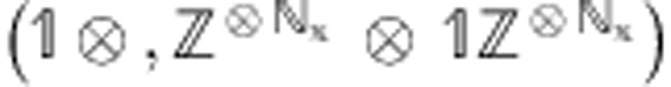 but breaks the symmetry under the action of 
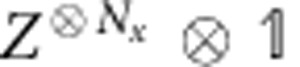
. Creating a physical excitation with flux quantum number 0 (string of identities) or 1 (string of *Z*'s) is manifested at the virtual level as a boundary state in the topologically trivial or, respectively, topologically non-trivial sector. That is, excitations with non-trivial flux correspond to domain-wall excitations at the level of the transfer matrix. The charge of the physical excitation can be measured as a charge difference between the ket and bra level of the boundary state using the 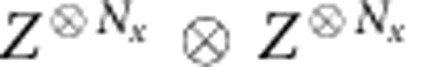 operator (which is a symmetry, that is, eigenvalue 1, for the fixed point subspace).

**Figure 7:**
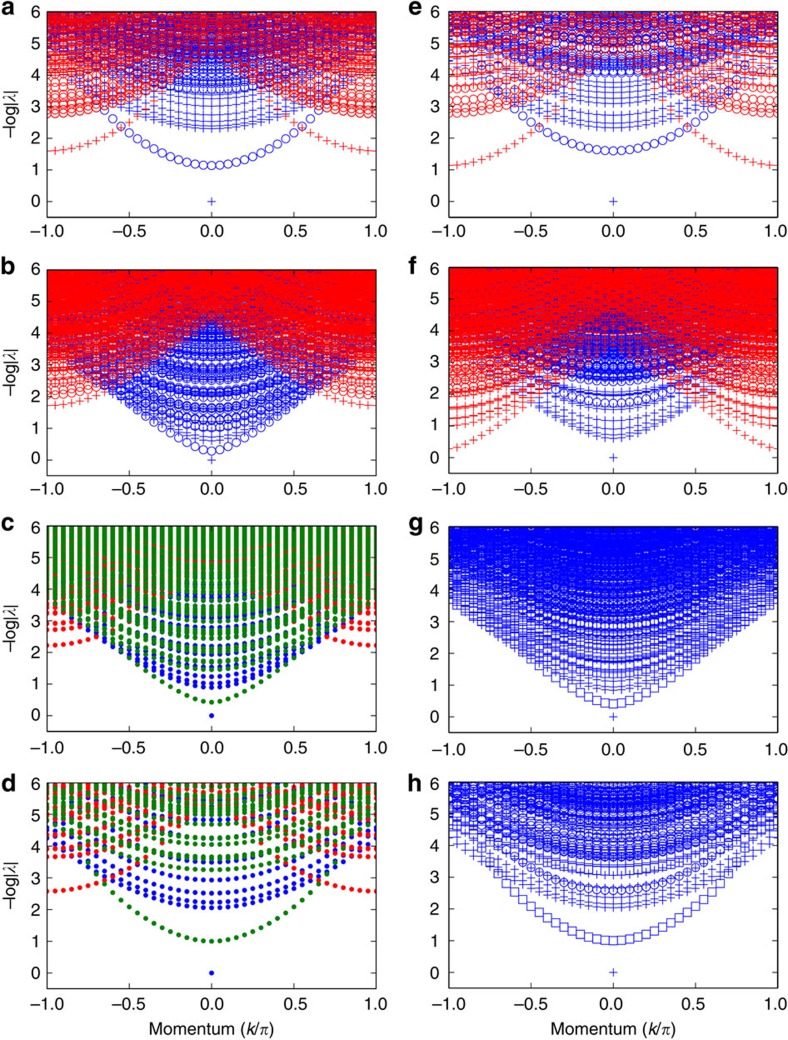
Transfer matrix spectra for the filtered toric code. We show (minus logarithm of) the eigenvalues *λ* of the transfer matrix as function of momentum *k*_*h*_ for the different points (*β*_*z*_,*β*_*x*_) indicated by the markers (**a**–**h**) in Fig. 6. In the topological phase (**a**,**b**,**e**,**f**), colours indicate topologically trivial (blue) or non-trivial (red) excitations (flux difference between ket and bra), while the symbol refers to equal (plus sign) or unequal (circle) charge between ket and bra level. As we move closer to the phase transition to the charge condensed phase (**a**→**b**), the gap of charge excitations decrease. In the charge condensed phase (**c**,**d**), charges can no longer be measured (dot symbols) and the *Z*^⊗*N*^⊗*Z*^⊗*N*^ symmetry is broken. This results in a new topologically non-trivial excitation (green) with a string in both ket and bra. The gap in this sector acts as string tension, indicating that flux excitations become confined, and increases as we move deeper into the trivial phase (**c**→**d**). Analogously, the gap of the flux excitations decreases when moving in the topological phase from **e** to **f** and eventually results in flux condensation. In the flux condensed phase (**g**,**h**), the full symmetry is restored and no domain-wall excitations of the transfer matrix exist, since they are equivalent to local excitations. In addition, charge can be measured in both ket and bra separately. The states with charge difference (circle) can have individual ket and bra charges +, − and −, + but remain degenerate. States with no charge difference can have ket and bra charges +, + (plus sign) or −, − (square). The gap in the latter sector acts as string tension between charges and thus indicates charge confinement; it increases when moving deeper into the trivial phase (**g**→**h**).

Following ref. 11, we can now probe the dispersion relation of the elementary excitations of the model by inspecting the spectrum of (minus logarithm of) the eigenvalues of the transfer matrix in the different sectors, for which we use the one-dimensional excitation ansatz of ref. 17. The result is illustrated for various values of *β*_*z*_ and *β*_*x*_ inside and outside the topological phase in Fig. 6. In the topological phase (plots (a), (b), (e) and (f)), the eigenvectors can be labelled by the charge difference between ket and bra (corresponding to the physical charge) and the absence or presence of a half-infinite string (corresponding to the physical flux). We can then relate the spectrum of the transfer matrix to the dispersion relation within the four physical topological sectors and recognize the charge and flux as elementary excitations (isolated branch). In the first column of Fig. 7, the gap in the charge sector closes, resulting in charge condensation and a phase transition to the trivial phase (plots (c) and (d)). Here the fixed point subspace breaks the full symmetry 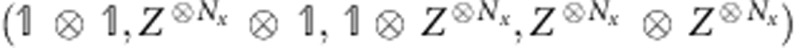 of the transfer matrix and the charge differences between ket and bra is no longer well defined. Correspondingly, we can create new topologically non-trivial excitations with a half-infinite string of *Z*'s in both ket and bra. That this sector has a gap Δ_*ZZ*_ indicates that physical flux excitations can no longer exist in isolation and must be confined to pairs, since their normalization goes down as exp(−Δ_*ZZ*_*L*) with *L* the length of the string. In the right column of Fig. 7, the gap in the flux sector closes, corresponding to physical flux condensation. This triggers a phase transition to the trivial phase and results at the virtual level in a unique fixed point with the full symmetry of the transfer matrix. Correspondingly, there are no more topologically non-trivial excitations and we can now measure individual charge numbers of the ket and the bra level. That there is a gap in the (−,−) sector with negative charge in ket and bra indicates that physical charge excitations can no longer exist in isolation and must be confined.

### Resonating valence bond state on the hexagonal lattice

**Figure 8:**
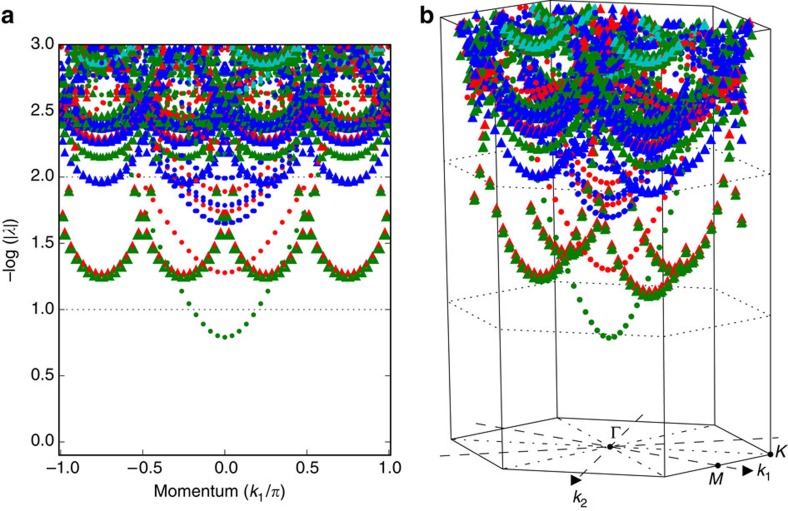
Transfer matrix spectrum of the resonating valence bond state on the Kagome lattice. In every sector with momentum *k*_1_, we obtain a set of complex eigenvalues *λ* of the transfer matrix. We interpret (minus the logarithm of) the magnitude of those eigenvalues as energy and obtain the plot in **a**. By also associating the phase of these eigenvalues to a momentum *k*_2_, we can plot this spectrum in the two-dimensional Brilluoin zone of the Kagome lattice, as in **b**. Colours indicate total spin *S* (red: 0, green: 1/2, blue: 1, cyan: 3/2) whereas markers indicates eigenvalues in the trivial (dot) or non-trivial (triangle) sector.

**Figure 9:**
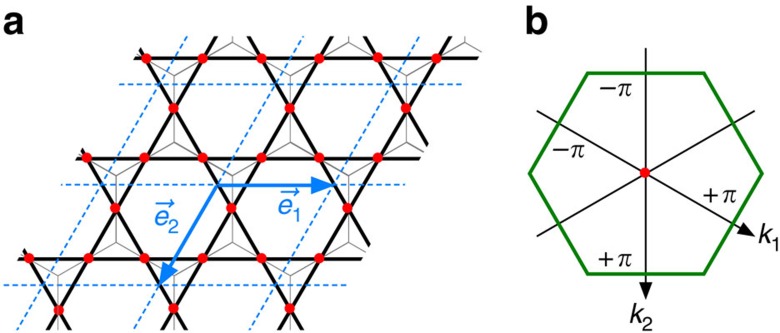
Kagome lattice and its brilluoin zone. We study the eigenvalues of the PEPS transfer matrix for the resonating valence bond state on the Kagome lattice. **a** depicts the Kagome lattice (black) with location of the spins (red dots) and indicates how these were blocked (blue lines) into a (distorted) square PEPS with lattice vectors 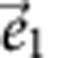 and 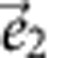 (blue arrows). **b** illustrates the corresponding Brillouin zone and indicates the oblique axis associated to the momenta *k*_1_∈(−*π*,+*π*) and *k*_2_∈(−*π*,+*π*) obtained from the PEPS calculation.

Finally, in Fig. 8 we present the spectrum of the transfer matrix for the resonating valence bond state^40^ on the Kagome lattice, for which the PEPS is also 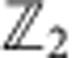-injective^41^,^42^. The Kagome lattice was blocked as illustrated in Fig. 9b, and the eigenvalues of the transfer matrix along the lattice vector 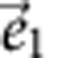 were computed, giving full access to the momentum *k*_1_. The phase of these eigenvalues can then be interpreted as momentum *k*_2_, which allows to map them to the Brillouin zone according to Fig. 9c. This clearly allows to extract the physical elementary excitations^43^,^44^, namely the spinons (*S*=1/2, no string) and visons (*S*=0, string), but also vison–spinon bound states (*S*=1/2, string), which seem to occur at energies lower than the vison energies. Finally, there are also an isolated branch of trivial spinless excitations (*S*=0, no string), which could be a bound state of either a vison pair or a spinon pair. For this model, the transfer matrix is a non-hermitian MPO and one can question the validity of an excitation ansatz based on a local perturbation on top of the fixed point (which is approximated as MPS), as this ansatz is only provably justified for the case of local Hamiltonians^14^. Numerical evidence for the validity of our results is provided in Supplementary Note 2 and Supplementary Figs 2 and 3.

## Discussion

We have illustrated how the eigenvalue spectrum of the one-dimensional (quantum) transfer matrix provides a holographic description of the dispersion relations of elementary excitations in the full two-dimensional quantum system. This holds true even in systems with topological order, where the elementary excitations are anyons. The presense of topological order gives rise to particular (virtual) symmetries of the transfer matrix. By carefully studying the manifestation of these symmetries in the fixed-point subspace, we were able to relate the different topological sectors of the physical excitations to corresponding topologically non-trivial symmetry sectors (domain walls) at the virtual level. This shows, in particular, that the existence of anyon excitations requires a particular type of symmetry breaking of the doubled virtual symmetry in the fixed-point subspace of the transfer matrix, whereas topological phase transitions give rise to a fixed-point subspace with a larger or smaller degeneracy.

While these results might be reminiscent of the closely related bulk-edge correspondence observed in chiral topological phases^45^,^46^, we would like point out the subtle differences. In the PEPS formalism, the properties of the edge states are determined by the fixed points of the transfer matrix^47^, whereas here we explicitly consider the complete (long-distance) spectrum of the transfer matrix. In addition, the framework for characterizing topological order in PEPS using MPOs, which is of central importance for our results, has so far only been made explicit for the non-chiral string net models, and it remains to be clarified how the recently discovered chiral PEPS^48^,^49^,^50^ fit within this framework.

This technique holds a powerful potential for studying fundamental questions of topological order and topological phase transitions. While we have studied transfer matrices originating from a tensor network representation of the ground state, the results presented in this paper should generalize to the full-quantum transfer matrix obtained from representing the ground state as an imaginary time-path integral. Whereas the exact path integral representation can have a gauge theory as virtual boundary, the PEPS truncation will eliminate the gauge degrees of freedom. Correspondingly, the local order parameter measuring the symmetry breaking transitions at the PEPS virtual level will map to a string operator in the temporal direction of the full-path integral.

## Additional information

**How to cite this article:** Haegeman, J. *et al.* Shadows of anyons and the entanglement structure of topological phases. *Nat. Commun.* 6:8284 doi: 10.1038/ncomms9284 (2015).

## Supplementary Material

Supplementary Information Supplementary Figures 1-3, Supplementary Notes 1-2 and Supplementary References
