# Supplementary material for: Shadows of anyons and the entanglement structure of topological phases
Source: Nat Commun. 2015 Oct 6;6:8284. doi: 10.1038/ncomms9284 (PMC4600714; doi:10.1038/ncomms9284)
Supplement: Supplementary Information — Supplementary Figures 1-3, Supplementary Notes 1-2 and Supplementary References [file ncomms9284-s1.pdf]

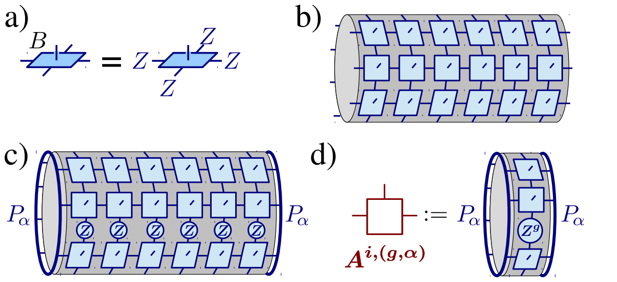

Supplementary Figure 1: **$\mathbb{Z}_2$ injective PEPS on the cylinder**. (a) Symmetry property of a $\mathbb{Z}_2$ injective PEPS tensor; (b) PEPS on the infinite cylinder; (c) Construction of different topological sectors; (d) Construction of the corresponding MPS tensor.

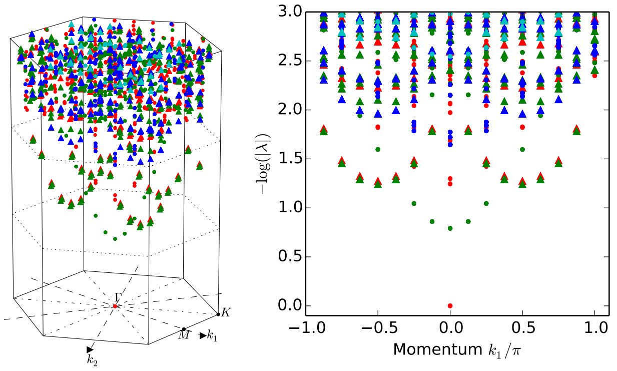

Supplementary Figure 2: **Transfer matrix spectrum of the RVB on the cylinder**. We plot the spectrum of the PEPS transfer matrix for the RVB state on a cylinder with circumference $N_x = 8$. Markers indicate $g - g' \mod 2 = 0$ (dots) or $g - g' \mod 2 = 1$ (triangles), whereas colors indicate the spin S of the excitation (red: $S = 0$, green: $S = 1/2$, blue: $S = 1$, cyan: $S = 3/2$). The value of $\alpha - \alpha'$ is superseded by the spin quantum number, since all integer spin excitations have $\alpha - \alpha' \mod 2 = 0$ and all half integer spin excitations have $\alpha - \alpha' \mod 2 = 1$.

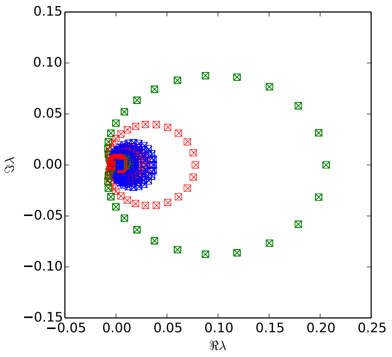

Supplementary Figure 3: **Alternative confirmation of the validity of the excitation ansatz**. Comparison of the eigenvalues of $(P\mathbb{T}P)^2$ (squares) with the eigenvalues of $P\mathbb{T}^2P$ (crosses), where P is the projector onto the subspace spanned by the variational excitation ansatz in the trivial sector. The fact that these points collapse for the eigenvalues λ of largest magnitude confirms that the variational subspace can accurately capture the eigenvectors of $\mathbb{T}$ corresponding to the largest $|\lambda|$ (smallest $-\log|\lambda|$).

Supplementary Note 1

We will first recapitulate explicitly how to construct the mixed transfer operator in the case of 1D systems. A Matrix Product State $|\psi\rangle = \sum_{s_1,\dots,s_L} \mathrm{tr}[A^{s_1}\cdots A^{s_N}]$ with topologically non-trivial excitations, this is, symmetry breaking, is described by block-diagonal tensors $A^s = \oplus_i A^{s,i}$. The symmetry broken ground states $|\psi_i\rangle$ are then described by the individual $A^{s,i}$, and domain wall excitations are obtained by switching between different i in the matrix product (alternatively, this can be achieved by attaching a string operator which transforms a product of $A^{s,i}$ to one of $A^{s,i'}$). In the overlap of the momentum superpositions of these domain walls, the mixed transfer operator $\mathbb{T}^i_{i'} = \sum_s A^{s,i} \otimes \bar{A}^{s,i'}$ appears between the ket and bra position of a domain wall between sectors i and i'. Its leading eigenvalue λ thus characterizes the correlation function of a pair of such excitations and can be used to infer the momentum $\arg(\lambda)$ and the quasi-energy $-\log|\lambda|$ of the minima of the dispersion.

One can additionally assign quantum numbers of non-broken symmetries to these excitations. To this end, consider a symmetry $|\psi\rangle = U_g^{\otimes L}|\psi\rangle$ (for simplicity, we restrict to finite groups $\mathsf{G} \ni g$). Then, it holds that $\sum_t (U_g)_{st} A^t = V_g^\dagger A^s V_g$ [1], where V_g inherits the algebraic structure of U_g (more precisely, it is an induced projective representation [2]). The quantum number of an excitation is then given by the quantum number of the corresponding left eigenvector of the (mixed) transfer operator with respect to $V_g \otimes \bar{V}_g$.

Let us now turn towards 2D cylindrical systems. Both the (perturbed) toric code and the resonating valence bond states discussed in the main text have a $\mathbb{Z}_2$-injective PEPS representation. The tensors then have a $\mathbb{Z}_2$ sym-

metry on the auxiliary indices, as illustrated in Supplementary Figure **1**(a). We consider states on an infinite cylinder with circumference N_x; the overall state is then obtained by arranging the tensors on the cylinder and contracting the auxiliary indices as in Supplementary Figure **1**(b). We denote the tensor obtained by blocking all tensors in one column (which yields an MPS) by A. The four minimally entangled topological sectors, corresponding to the four different quasi-particles, are obtained by *(i)* placing a string of Z^g ($g = 0, 1$) along the cylinder axis and *(ii)* choosing boundary conditions supported in the irreducible representations $\alpha = \pm 1$ of $Z^{\otimes N_x}$, as depicted in Supplementary Figure **1**(c). By interpreting this system as a one-dimensional chain, we can then use the symmetry of the PEPS tensor to construct four independent blocks of the MPS tensor $A^{i,(g,\alpha)}$ by blocking a column as in Supplementary Figure **1**(d). The corresponding mixed transfer operator is given by

$$\mathbb{T}_{g,\alpha}^{g',\alpha'} = \sum_s A^{s,(g,\alpha)} \otimes \bar{A}^{s,(g',\alpha')} \ . \qquad (1)$$

As explained in the main text (see Fig. 4), the value of g and g' determines the presence of a string in ket and/or bra of the corresponding domain wall excitations of the normal transfer matrix. However, in the thermodynamic limit of the topological phase, the symmetry of the fixed point under $Z^{\otimes N_x} \otimes Z^{\otimes N_x}$ ensures that the results for $(g, g') \to (g + 1 \mod 2, g' + 1 \mod 2)$ are exactly degenerate, so that the results only depend on $g - g' \mod 2$. In addition, because the fixed point subspace of the transfer matrix breaks the $Z^{\otimes N_x} \otimes \mathbb{1}$ symmetry, only the difference $\alpha - \alpha' \mod 2$ can be measured. While it is possible to build fixed points with well defined quantum numbers for α and α' individually, the pairs (α, α') and $(\alpha + 1 \mod 2, \alpha' + 1 \mod 2)$ will also be exactly degenerate, and only depend on the difference $\alpha - \alpha' \mod 2$. Note that on a cylinder with finite circumference N_x, the degeneracy will only manifest itself up to exponentially small corrections in N_x.

By blocking the cylindrical PEPS into an MPS, the translation invariance in the x direction (transversal direction) becomes an on-site symmetry, and we can still label the eigenvectors of the transfer matrix with the corresponding quantum numbers. To this end, we need to determine the virtual action τ of the physical translation operator T_x; the momenta are then given by the eigenvalues of $\tau \otimes \bar{\tau}$ evaluated on the corresponding eigenvector of $\mathbb{T}_{g,\alpha}^{g',\alpha'}$. This can be done by considering Fig. 5 of the main text for this particular case. As one can see, the action of τ corresponds to a translation of the virtual indices. However, if a string of Z's is present (i.e. $g = 1$), this string is moved as well, and restoring it at its original position requires an additional multiplication to be performed by Z, i.e., $\tau = T \cdot Z_1$, where T is the regular translation operator and Z_1 acts on the spin next to the location of the string. This leads to an important consequence for the labeling of momenta. We have that $\tau^{N_x} = (Z^g)^{\otimes N_x}$, which implies that τ^{N_x} has eigenvalue -1 when both $g = 1$ and $\alpha = -1$. Correspondingly, $(\tau \otimes \bar{\tau})^{N_x} = Z^{\otimes N_x} \otimes Z^{\otimes N_x} = \pm 1$, with eigenvalue -1 corresponding to the sectors $(g = 1 \neq g', \alpha = 1)$, $(g \neq 1 = g', \alpha' = 1)$ or $(g = g' = 1, \alpha \neq \alpha')$. In those sectors, the eigenvalues e^{ik} of $\tau \otimes \bar{\tau}$ are shifted by half a spacing, i.e., $k_x = 2\pi(n + \frac{1}{2})/N_x$, with $n = 0, \ldots, N_x - 1$. This can be checked for the data presented in Supplementary Figure **2** for $N_x = 8$. The fact that this results in smooth dispersion relations for $2N_x$ instead of N_x different momenta is truly remarkable.

Supplementary Note 2

We can also compare the exact diagonalization results of Supplementary Figure **2** with the results obtained using the variational excitation ansatz in the limit $N_x \to \infty$, as presented in Fig. 8 of the main text. These results match perfectly, where of course the results in the main text do not suffer from finite size effects (but perhaps from finite D effects in the MPS approximation) and can have an arbitrary resolution in momentum space, since all quantum numbers $k_1 \in [-\pi, +\pi)$ are available in the thermodynamic limit. The variational excitation ansatz defines, for every momentum slice, a linear subspace of the Hilbert space. If P_k denotes the orthogonal projector onto this subspace, we are essentially computing the eigenvalues of $P_k \mathbb{T} P_k$ instead of the eigenvalues of $\mathbb{T}$, which could be very different if the variational subspace would not be able to accurately capture exact eigenvectors of $\mathbb{T}$. As a further confirmation, we compare in Supplementary Figure **3** the eigenvalues of $(P\mathbb{T}P)^2$ with the eigenvalues of $P\mathbb{T}^2P$, where P is the projector onto the total excitation subspace $P = \int \mathrm{d}k P_k$.

Supplementary references

[1] Perez-Garcia, D., Wolf, M. M., Sanz, M., Verstraete, F. & Cirac, J. I. String Order and Symmetries in Quantum Spin Lattices. *Phys. Rev. Lett.* **100**, 167202 (2008).

[2] Schuch, N., Pérez-García, D. & Cirac, I. Classifying quantum phases using matrix product states and projected entangled pair states. *Phys. Rev. B* **84**, 165139 (2011).
